# Secondary Lower-Motor-Neuron Facial Palsy Revealing Buccal Squamous Cell Carcinoma with Parotid Duct Obstruction and Perineural Invasion

**DOI:** 10.3390/diagnostics16142289

**Published:** 2026-07-22

**Authors:** Yu-Cheng Chu, Ping-Yi Lin, Wen-Chih Huang

**Affiliations:** 1 Department of Neurology, Far Eastern Memorial Hospital, New Taipei City 220, Taiwan; 2 Graduate Institute of Management, Chang Gung University, Taoyuan City 333, Taiwan; 3 Department of Dentistry, Far Eastern Memorial Hospital, New Taipei City 220, Taiwan; rhyne.lin@gmail.com; 4 School of Dentistry, National Taiwan University, Taipei 100, Taiwan; 5 Department of Anatomical Pathology, Far Eastern Memorial Hospital, New Taipei City 220, Taiwan; pathology.taipei@gmail.com

**Keywords:** Bell’s palsy, lower-motor-neuron facial palsy, facial nerve palsy, squamous cell carcinoma, buccal mucosa, oral cancer, perineural invasion, parotid duct obstruction, magnetic resonance imaging

## Abstract

Peripheral facial palsy is often attributed to idiopathic Bell’s palsy, but secondary structural causes should be considered when local red flags are present. A 61-year-old man with a 40-pack-year smoking history and former betel quid chewing presented with a verrucous-appearing mass involving the left oral commissure and buccal mucosa, intermittent purulent discharge from the lesion, progressive left facial swelling, and ipsilateral lower-motor-neuron facial palsy with lagophthalmos. Magnetic resonance imaging demonstrated a left buccal/oral-cavity lesion with ipsilateral parotid duct obstruction. He underwent tracheostomy, wide excision, left supraomohyoid neck dissection, and radial forearm free-flap reconstruction. Pathology confirmed squamous cell carcinoma, pT2N0, cM0 (stage II), with perineural invasion; surgical margins were negative, and lymphovascular invasion was not identified. At follow-up, wound healing was satisfactory and purulent discharge had resolved, but lower-motor-neuron facial palsy persisted; adjuvant radiotherapy was recommended. This case emphasizes that lower-motor-neuron facial palsy with an oral mass, purulent discharge, facial swelling, or salivary-duct obstruction should prompt careful oral examination and head-and-neck imaging.

**Figure 1:**
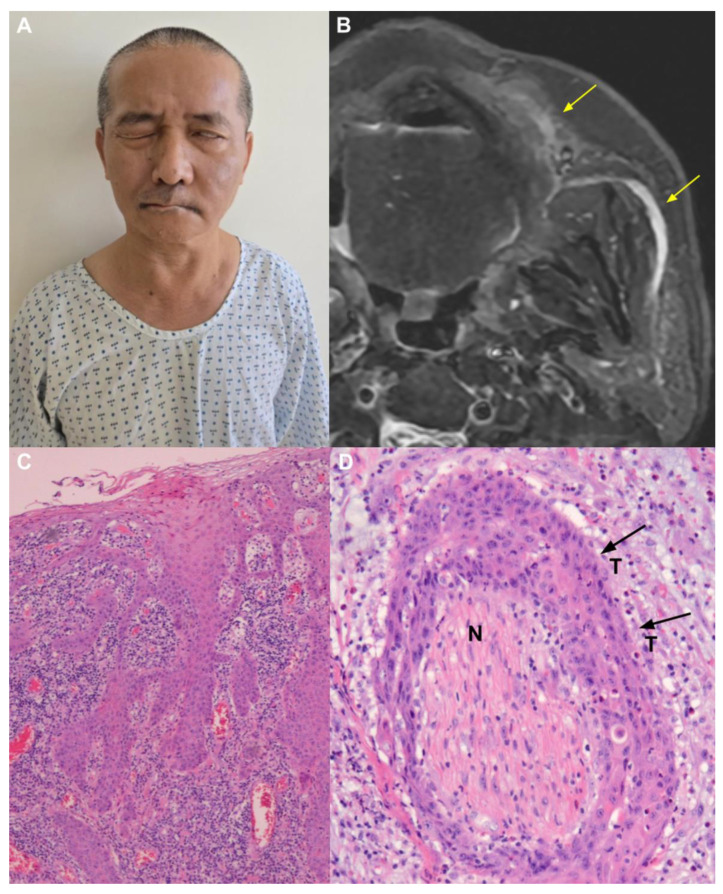
Buccal squamous cell carcinoma presenting with secondary lower-motor-neuron facial palsy, parotid duct obstruction, and perineural invasion. (**A**) Clinical photograph obtained during attempted eye closure demonstrates left lower-motor-neuron facial palsy with lagophthalmos. (**B**) Axial T2-weighted short tau inversion recovery neck magnetic resonance image, cropped to the left buccal-parotid region, shows high-signal soft-tissue change in the left buccal/oral-cavity region (upper arrow) and dilatation of the left parotid duct secondary to tumor-related obstruction (lower arrow); the patient’s left side appears on the right side of the image. (**C**) Hematoxylin and eosin staining of the main tumor demonstrates invasive squamous cell carcinoma involving the buccal mucosa (original magnification, ×40). (**D**) Higher-power hematoxylin and eosin staining demonstrates perineural invasion: tumor cells (T, arrows) encircle or track along a nerve bundle (N) (original magnification, ×100). Final surgical pathology showed moderately differentiated conventional squamous cell carcinoma of the left buccal mucosa, negative surgical margins, no lymphovascular invasion, negative cervical lymph nodes, and microscopic perineural invasion. The pathologic classification was pT2N0, with no clinical evidence of distant metastasis (cM0; stage II). A 61-year-old man presented with several weeks of a verrucous-appearing mass involving the left oral commissure and buccal mucosa, intermittent purulent discharge from the oral lesion, progressive left facial swelling, and ipsilateral lower-motor-neuron facial palsy (Figure 1A,B). His social history included a 40-pack-year smoking history and former betel quid chewing; alcohol use was denied. In the Taiwanese clinical context, tobacco exposure and betel quid chewing are particularly relevant risk factors for oral squamous cell carcinoma [1,2]. The differential diagnosis of lower-motor-neuron facial palsy included idiopathic Bell’s palsy, Ramsay Hunt syndrome, otitis or parotid inflammatory disease, brainstem stroke, skull-base or parotid malignancy, and perineural tumor spread. The local oral findings, purulent discharge, facial swelling, and salivary-duct obstruction argued against idiopathic Bell’s palsy, which remains a diagnosis of exclusion [3]. The clinically affected nerve was the left facial nerve; however, MRI demonstrated the buccal lesion and ipsilateral parotid duct obstruction, but showed no definite macroscopic enhancement, enlargement, or involvement of the main facial nerve trunk or facial canal. Therefore, the mechanism was interpreted cautiously as tumor-related neural involvement, supported by microscopic perineural invasion and adjacent inflammatory or edematous change, rather than proven macroscopic perineural spread along the named facial nerve. This distinction is important because microscopic perineural invasion and radiologically evident perineural spread are related but not synonymous. Perineural invasion in oral squamous cell carcinoma is an adverse histopathological feature and may involve small intratumoral or peritumoral nerves (Figure 1C,D) [4,5], whereas perineural spread refers to macroscopic extension along larger named nerves that may be visible on an MRI and may correlate with cranial neuropathy [6,7]. In the present case, the histopathological finding of perineural invasion supports tumor-associated neural involvement but does not by itself prove direct facial nerve trunk invasion. The patient underwent tracheostomy, wide excision of the left buccal tumor, left supraomohyoid neck dissection, and left radial forearm free-flap reconstruction. At approximately six weeks after surgery, wound and flap healing were satisfactory and purulent discharge had resolved; however, left lagophthalmos and lower-motor-neuron facial palsy persisted. Because final pathology showed perineural invasion despite negative margins and the absence of lymphovascular invasion, adjuvant radiotherapy at 64 Gy in 32 fractions was recommended, and PET/CT was planned to evaluate occult nodal or distant disease and assist radiotherapy target delineation.

## Data Availability

The data underlying this article are not publicly available because they contain potentially identifiable clinical information and medical images. De-identified data may be made available from the corresponding author upon reasonable request and with appropriate institutional approval.
